# Increased Levels of the Parkinson’s Disease-Associated Gene ITPKB Correlate with Higher Expression Levels of α-Synuclein, Independent of Mutation Status

**DOI:** 10.3390/ijms24031984

**Published:** 2023-01-19

**Authors:** Francesca Di Leva, Michele Filosi, Lisa Oyston, Erica Silvestri, Anne Picard, Alexandros A. Lavdas, Evy Lobbestael, Veerle Baekelandt, G. Gregory Neely, Peter P. Pramstaller, Andrew A. Hicks, Corrado Corti

**Affiliations:** 1 Institute for Biomedicine, Eurac Research, Affiliated Institute of the University of Lübeck, 39100 Bolzano, Italy; 2 Charles Perkins Centre and School of Life and Environmental Sciences, The University of Sydney, Sydney, NSW 2006, Australia; 3 Laboratory for Neurobiology and Gene Therapy, Department of Neurosciences, Leuven Brain Institute, KU Leuven, 3000 Leuven, Belgium

**Keywords:** α-synuclein, A53T, A30P, ITPKB, Parkinson’s disease, PD cortex, *Drosophila*, *IP3K2*

## Abstract

Autosomal dominant mutations in the gene encoding α-synuclein (*SNCA*) were the first to be linked with hereditary Parkinson’s disease (PD). Duplication and triplication of *SNCA* has been observed in PD patients, together with mutations at the N-terminal of the protein, among which A30P and A53T influence the formation of fibrils. By overexpressing human α-synuclein in the neuronal system of *Drosophila*, we functionally validated the ability of *IP3K2*, an ortholog of the GWAS identified risk gene, Inositol-trisphosphate 3-kinase B (*ITPKB*), to modulate α-synuclein toxicity in vivo. *ITPKB* mRNA and protein levels were also increased in SK-N-SH cells overexpressing wild-type α-synuclein, A53T or A30P mutants. Kinase overexpression was detected in the cytoplasmatic and in the nuclear compartments in all α-synuclein cell types. By quantifying mRNAs in the cortex of PD patients, we observed higher levels of *ITPKB* mRNA when *SNCA* was expressed more (*p* < 0.05), compared to controls. A positive correlation was also observed between *SNCA* and *ITPKB* expression in the cortex of patients, which was not seen in the controls. We replicated this observation in a public dataset. Our data, generated in SK-N-SH cells and in cortex from PD patients, show that the expression of α-synuclein and ITPKB is correlated in pathological situations.

## 1. Introduction

Parkinson’s disease (PD) is a complex, progressive neurodegenerative disorder which affects 1% of the population over 60 years of age [1]. Bradykinesia in combination with resting tremors and/or rigidity represent the motor symptoms required for PD diagnosis. In addition, non-motor symptoms, which appear earlier, are observed in many patients. These include insomnia, constipation, depression, anxiety and hallucinations [1,2]. From a pathological perspective, PD patients show a progressive loss of dopaminergic neurons in the substantia nigra *pars compacta*, while the surviving neurons contain Lewy Bodies (LB) and Lewy Neurites (LN), mainly composed of insoluble aggregates of phosphorylated α-synuclein. Genetic factors account for 5–10% of inherited forms of PD [1]. Initially, mutations and amplifications of the *SNCA* gene, that encodes α-synuclein, were identified in familial PD [3,4,5]. Based on clinical data, patients carrying a *SNCA* gene triplication show the most severe phenotype and have earlier disease onset compared with those carrying a duplication; the same was observed for patients carrying missense mutations when compared to idiopathic PD [6]. To date, A53T [4] and A30P [7] are among the few dominant missense mutations identified in α-synuclein. They are both located in the N-terminal domain of the protein and influence α-synuclein aggregation. In the presence of A53T, aggregation speed seems to be more rapid compared to the wild-type [8] while the effect of A30P is still not clear [8,9,10]. Clinically, members of the family carrying A30P show a delayed onset of the disease accompanied by cognitive impairment [7], while A53T carriers display an earlier onset, rapid progression and the presence of cognitive, psychiatric and autonomic impairment of different severities [11].

Since initial reports in 1996, mutations in the *SNCA* gene have been associated with PD, but people carrying the same mutation may show different degrees of severity in the phenotype, as is the case with A53T, suggesting the existence of genetic modifiers. Recently, the gene that encodes the ubiquitously expressed Inositol-trisphosphate 3-kinase B (*ITPKB*) was associated with Parkinson’s disease (PD) in a genome-wide association study [12]. ITPKB phosphorylates inositol 1,4,5-trisphosphate (IP3) and converts it into inositol 1,3,4,5 tetrakisphosphate (IP4) via a Ca^2+^/Calmodulin-dependent mechanism [13]. The production of IP4 reduces the release of Ca^2+^ from the endoplasmic reticulum (ER) storage compartment into the cytoplasm via IP3 receptors (IP3R) [14]. Initially, *Itpkb* was characterized in mice where it drives the maturation of T-lymphocytes, during CD3/CD4 double-stage-positive selection [15,16], as well as the development of B-lymphocytes [17]. ITPKB has also been associated with Alzheimer’s disease (AD) by a study studies showing increased levels of *ITPKB* mRNA in the frontal cortex of AD patients [18]. In AD mouse models, an increased level of Itpkb enhances the formation of amyloid-β peptides and the phosphorylation of tau, hallmarks of the disease, via IP4/ERK1/2 signaling [19,20]. Another actor in this pathway is miR-132, which is downregulated in mice and leads to the increase of Itpkb and consequently to the increased activity of BACE1—the enzyme that produces amyloid-β peptides—and tau phosphorylation [20]. Of note, miR-132 is downregulated in the brains of AD patients. Finally, by targeting *Itpkb* with miR-711, it is possible to reduce inflammation and tau phosphorylation in a traumatic brain injury mouse model [21].

In primary mouse cortical neurons treated with preformed fibrils, the production of IP4 via ITPKB reduces Ca^2+^ upload into the mitochondria, after ER release, thus protecting cells from the overproduction of ATP and ROS generation, which induce the accumulation of α-synuclein aggregates [22]. In addition to this work, we also demonstrated in a recently characterized new *C. elegans* model for PD (*erals1*), which is engineered to express human α-synuclein, that the loss of function of *Ife-2*, the worm ortholog of *ITPKB* increases the formation of α-synuclein inclusions in the dendrites of adult animals [23].

Here, we initially demonstrate that by knocking down *IP3K2*, the closest fly ortholog of *ITPKB*, in *Drosophila* overexpressing human α-synuclein, we could rescue α-synuclein-induced PD-related phenotypes (geotaxis and lifespan). We then analyzed ITPKB expression in human neuroblastoma cell lines in which wild-type or mutated *SNCA* is overexpressed and also determined mRNA level in postmortem cortex tissue originating from PD patients We found that a direct correlation in the expression of *SNCA* and *ITPKB* exists in both disease models thus suggesting that ITPKB may play a role in the pathogenicity of *SNCA* overexpression.

## 2. Results

### 2.1. Neuronal Knockdown of IP3K2 Increases Lifespan and Climbing of Transgenic Flies Expressing Human αSyn in the Brain

From a functional screen of PD risk genes in the *C. elegans* PD model *eraIs1*, we previously identified *lfe-2*, a sole ortholog of three human *ITPKs* (the neuronal *ITPKA*, the ubiquitously expressed *ITPKB* and the glial *ITPKC*), as a modulatory gene of α-synuclein-induced neurodegeneration. Since loss-of-function mutations in *lfe-2*, which increased formation of α-synuclein inclusions in the dendrites of adult animals [23], also reduced the brood size and fertility of the animals, we suspected that complete inactivation of ITPK activity does not accurately represent the pathological role of ITPKB in PD. Therefore, we investigated the role of ITPKB activity in a fruit fly PD model system. To evaluate the potential role of ITPKB in modifying α-synuclein toxicity in vivo, we expressed human α-synuclein in the nervous system of transgenic *Drosophila melanogaster* (*elavGal4*; UAS-h-αSyn). This model exhibits a progressive loss of motor function and shortened lifespan. UAS has no effect on climbing activity.

**Figure 1:**
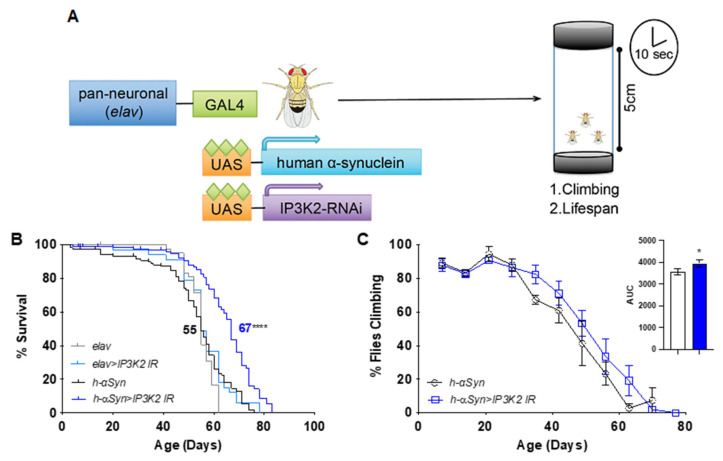
Neuronal knockdown of *IP3K2* increases lifespan and climbing in *h-αSyn* expressing flies. (**A**) Neuronal knockdown (*elavGal4*) of *ITPKB* ortholog, *IP3K2* was tested for its modification of *h-αSyn* phenotypes, climbing and lifespan. (**B**) Knockdown of *IP3K2* increased lifespan in an *h-αSyn*-dependent manner. (**C**) Climbing ability of *IP3K2* knockdown (*h-αSyn > IP3K2 IR*) was slightly increased compared to the PD model (*h-αSyn*; N = 3; each *n* ≥ 40 animals). Data represent mean ± SEM. *p*-values calculated using log-rank Mantel-Cox test (**B**) and area under the curve analysis (**C**). * *p* < 0.05; **** *p* < 0.0001. *elav* = *elavGal4/+*; *+/+*; *UAS-mCD8GFP/+*. *elav > IR = elavGal4/+*; *UAS-IR/+*; *UAS-mCD8GFP/+*. *h-αSyn* = *elavGal4/+*; *+/+*; *UAS-h-αSyn/+. h-αSyn > IR* = *elavGal4*; *UAS-IR/+*; *UAS-h-αSyn*.

In *Drosophila melanogaster*, the human gene *ITPKB* has two orthologs, *IP3K1* and *IP3K2*, of which *IP3K2* exhibits greater homology to *ITPKB* (DIOPT score 11) and is more evolutionarily related to vertebrate ITPKs. Neural-specific knockdown of IP3K2 significantly increased survival in an h-αSyn expressing flies, extending median lifespan by 12 days (*p* < 0.0001; Figure 1A,B).

Targeting *IP3K2* alone had no significant effect, suggesting that the effect of *IP3K2* knockdown is h-αSyn-dependent (*p* = 0.0635; Figure 1B). Moreover, an increase in the climbing ability of 11% was also observed in h-αSyn-expressing flies with *IP3K2* knockdown (AUC *p* = 0.0476; Figure 1C). We interpret this data to demonstrate that there is a functional interaction in vivo between α-synuclein and ITPKB levels and that modulation of ITPKB may mitigate the negative consequences of mis-regulated α-synuclein.

### 2.2. Wild-Type or Mutated α-Synuclein Overexpression in SK-N-SH Cells Increases ITPKB Level

Since the in vivo data from PD invertebrate model system showed a modulatory effect of ITPKB activity in α-synuclein-dependent pathology, we determined ITPKB levels in human PD cellular model systems, where no data are currently available on the effect that mutations in *SNCA* gene may have on ITPKB expression or function. As first step, we ascertained if any difference exists in ITPKB expression when α-synuclein or its pathological isoforms are overexpressed. We used previously characterized SK-N-SH neuroblastoma cells stably overexpressing, in both mRNA and protein forms, α-synuclein wild-type (wt) or A53T or A30P mutated isoforms [24]. While no inclusions or aggregations of α-synuclein are observed in these cells, a α-synuclein positive staining is present throughout the cells [24], and thus this model can still be informative in revealing altered pathological mechanisms at early stages, before aggregates are formed. 

**Figure 2:**
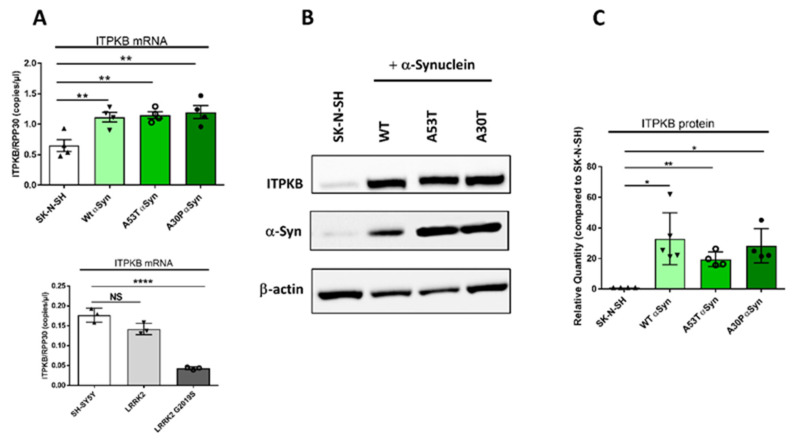
The ITPKB level is increased in SK-N-SH cell lines overexpressing wild-type or mutated A53T or A30P α-synuclein. SK-N-SH cells stably expressing wild-type (wt) or mutated A53T or A30P α-Synuclein were generated in house [24]. (**A**) Bar graphs represent *ITPKB* mRNA quantification in SK-N-SH cells or SK-N-SH cells overexpressing wild-type α-synuclein (wt) or A53T/A30P isoforms (top); *ITPKB* mRNA was also quantified in SH-SY5Y cells, and its level was compared with cells overexpressing LRRK2 or LRRK2 G2019S (bottom). N = 3–4 independent experiments were performed (**B**) ITPKB representative Western blot of 4–5 independent experiments. (**C**) Bar graphs represent ITPKB protein quantification in SK-N-SH cells or SK-N-SH cells overexpressing wild-type α-synuclein (wt) or A53T/A30P isoforms. N = 4–5 independent experiments were performed. Means ± SEM are shown. Statistical significance was calculated using a 1-way ANOVA with Bonferroni’s correction for multiple comparisons (ddPCR) or the Student’s *t*-test with Welch’s correction for unequal distribution (western blot). NS not significant, * *p* ≤ 0.05, ** *p* ≤ 0.01. **** *p* ≤ 0.0001.

We measured *ITPKB* mRNA and protein level in these cells and compared their levels with mock-transfected SK-N-SH control cell lines (Figure 2). 

To evaluate transcription in the cells, droplet digital PCR experiments were performed using a commercial assay specific for *ITPKB*. To normalize the expression among different samples, *RPP30* was selected as a housekeeping gene. When α-synuclein is overexpressed, *ITPKB* transcription is significantly enhanced by 65%, 61% and 79% for wt, A53T and A30P SNCA isoforms, respectively (Figure 2A, top panel). No clear differences in amounts of *ITPKB* expression were induced by specific α-synuclein isoforms (Figure 2A, top panel). These results may indicate that either the induced expression of *ITPKB* is not influenced by individual mutations in α-synuclein, or that if there is a difference in the individual mutation effect, this is masked by a general α-synuclein influence on the *ITPKB* expression effect. To assess the specificity of the α-synuclein-dependent ITPKB increase, we used SH-SY5Y neuroblastoma cells, in which LRRK2 or its most widespread mutant (G2019S) were expressed. LRRK2 is one of the most frequent risk factors for familial and idiopathic PD and the mutation G2019S leads to hyperactivation of the kinase activity. In SH-SY5Y cells, G2019S-LRRK2 overexpression leads to the accumulation of endogenous phosphorylated α-synuclein and the formation of structures similar to cytoplasmatic inclusions, which are not observed when wild-type LRRK2 is overexpressed [25]. In this cell model, wild-type LRRK2 does not appear to influence the *ITPKB* mRNA level, while the hyperactivated protein does significantly reduce the *ITPKB* transcript level to approximately 25% of that compared to SH-SY5Y- or LRRK2-overexpressing cells (Figure 2A, bottom panel). We conclude therefore that *ITPKB* expression is specifically increased in this cell model in the presence of an α-synuclein insult and that its increase can be differently modulated in relation to PD-causing mutations, which is potentially interesting for determining the direction of effect for possible therapeutic reasons. 

We then analyzed by Western blot if α-synuclein overexpression induced changes in *ITPKB* mRNA, and whether these changes are also reflected at the protein level. Proteins were extracted from SK-N-SH control cells or those overexpressing wild-type or mutated α-synuclein and analyzed by SDS-PAGE and Western blotting. We used a specific antibody to stain ITPKB while β-actin was used as a loading control (representative image in Figure 2B). In line with ddPCR data, we measured significant increases of 33%, 20% and 28% of ITPKB levels when α-synuclein or A53T/A30P isoforms were overexpressed compared to the control cells, respectively (Figure 2C). As was reported above for mRNA, and as expected, increased α-synuclein overexpression is mirrored by an induced increase in ITPKB protein, irrespective of mutation status. 

Thus, we conclude that in SK-N-SH cells overexpressing any forms of α-synuclein, a concomitant induced increase in *ITPKB* expression and protein is observed.

### 2.3. ITPKB Is Increased in Both Nucleus and Cytoplasm in SK-N-SH Cells Expressing Wild-Type or Mutated α-Synuclein

**Figure 3:**
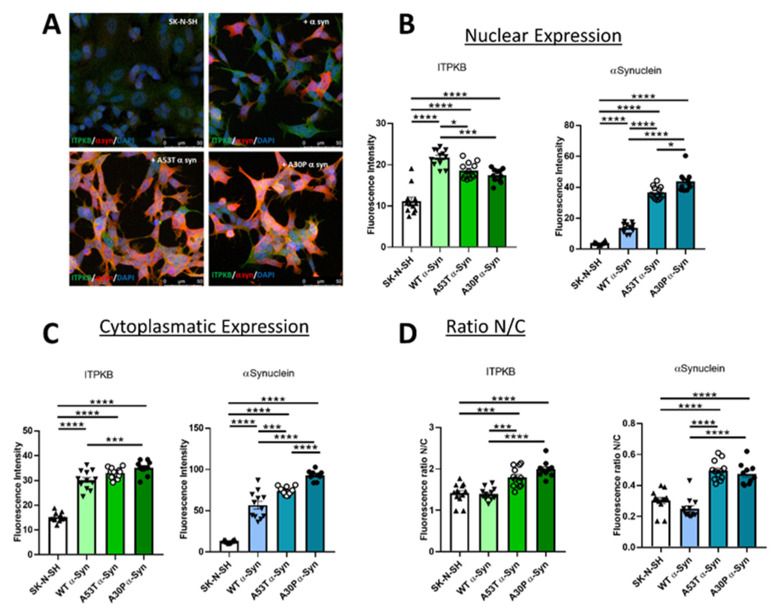
The ITPKB level is increased in the nucleus and in the cytoplasm of SK-N-SH cell lines overexpressing wild-type or mutated A53T or A30P α-synuclein. (**A**) Representative images of SK-N-SH cells stained for ITPKB (green), α-synuclein (red) and DAPI (blue). Wild-type SK-N-SH (top, left) or SK-N-SK cells transfected with a plasmid carrying wild-type (top, right), A53T (bottom, left) or A3 (bottom, right) mutants are shown. Scale bar = 50 µm. (**B**) Bar graphs represent ITPKB (left) or α-synuclein (right) quantification in the nucleus. (**C**) Bar graphs show ITPKB (left) or α-synuclein (right) quantification in the cytoplasm (**D**) Bar graphs represent the ratio between nuclear (N) and cytoplasmatic (**C**) quantification for ITPKB (left) or α-synuclein (right). N = 10–12 different fields were acquired. Mean ± s.e.m. are shown. Statistical significance was calculated using a 1-way ANOVA with Bonferroni’s correction for multiple comparisons. * *p* ≤ 0.05, *** *p* ≤ 0.001. **** *p* ≤ 0.0001.

Given the observed α-synuclein induced increase in *ITPKB*, we also ascertained its cellular localization, to determine if increases were widespread, or confined to specific cellular compartments. ITPKB was observed in different compartments in the cells (cytoplasm, nucleus and ER) and therefore we looked at whether amplification or mutations in the *SNCA* gene can differently influence the kinase localization eventually altering calcium homeostasis in local cellular environments. We prioritized the analysis of the ITPKB nucleus-to-cytoplasm diffusion because it has been shown that the localization of ITPKB to the plasma membrane is necessary to activate Ca^2+^-dependent transcription in dendritic cells following an inflammatory stimulus [26]. ITPKB contains an F-actin binding domain, a nuclear export and a nuclear localization signal at its N-terminus, which allow the shuttling of the kinase between the two compartments [27]. SK-N-SH cells or SK-N-SH cells overexpressing wild-type, A53T and A30P α-synuclein were stained for ITPKB and α-synuclein and imaged (representative staining for all samples is reported in Figure 3A).

We quantified ITPKB and α-synuclein in the nucleus and in the cytoplasm, separately. We first evaluated ITPKB expression in the nuclear compartment in all the samples and found that, compared to SK-N-SH cells, its expression is increased by 1.9-, 1.7- and 1.6-fold in the presence of wild-type, A53T or A30P α-synuclein overexpression, respectively (Figure 3B, left panel). We also observed a significant increase of ITPKB, compared to SK-N-SH cells, in the cytoplasm that was similar to what is observed in the nucleus in the presence of wild-type α-synuclein (two-fold) and was even higher in the presence of A53T or A30P mutants (2.2- and 2.3-fold, respectively, Figure 3C, left panel). We confirmed also that α-synuclein is expressed at higher levels in both compartments in α-synuclein-overexpressing samples, compared to naïve cells (3.5, 9.3 and 11.1-fold in the nucleus for α-synuclein wt, A53T and A30P respectively Figure 3B. 4.3, 5.6 and 7-fold in the cytoplasm for α-synuclein wt, A53T and A30P respectively Figure 3C, right panels). As reported before [24], cells overexpressing mutant α-synuclein show higher levels of the protein in both compartments, especially the A30P isoform. 

The effect on induced ITPKB levels in wild-type and mutated α-synuclein-expressing samples is significantly different, even though they are increased in both cell types, so we determined whether a change in the nucleus/cytoplasm ratio would be observed. We observed no difference between the distribution in these compartments in naïve and in wild-type α-synuclein cells (Figure 3D, left panel). However, in presence of the A53T and A30P mutations, this ratio is higher compared to the SK-N-SH cells or cells expressing wild-type α-synuclein (Figure 3D, left panel), because most of the increased ITPKB was found in the cytoplasm and less was found in the nucleus when compared to cells expressing wild-type α-synuclein (Figure 3B,C, left panels). In the case of α-synuclein, the ratio between nucleus and cytoplasm is also not altered in SK-N-SH, while it is higher in the presence of the mutations (Figure 3D, right panel). As already reported [28,29], and opposite to what is observed for ITPKB, in this case, A53T and A30P mutants are more expressed in the nucleus than in the cytoplasm (Figure 3B,C, right panels) while no difference with naïve cells was observed when wild-type α-synuclein was overexpressed. 

Globally, we interpret this data to confirm that in the presence of overexpressed α-synuclein, ITPKB levels are increased and that there may be a differential effect on induced expression levels, as observed for the A53T and A30P mutant isoforms. Indeed, in the presence of mutant α-synuclein isoforms, a change in the cellular distribution of ITPKB can occur, such that ITPKB is found more in the cytoplasm, which may lead to increased alterations in calcium homeostasis in the cytoplasm of carriers of these mutations.

### 2.4. ITPKB and SNCA Expression Is Also Correlated in the Cortex of Patients with Parkinson’s

To investigate whether the α-synuclein-induced increase of ITPKB expression also occurs in PD patients, we extracted RNA from the cortex of 11 PD patients and 15 healthy controls (mean age: 79.1 ± 5.9 and 73.9 ± 7.3 years at the age of death for patients and controls, respectively; demographic description and clinical records are given in Table 1).

Patients in this cohort demonstrated PD with or without the presence of Lewy bodies and, in some cases (*n* = 5), also Alzheimer’s disease was clinically annotated. Unfortunately, no genetic information on mutation carrier status is available for the patient cohort. Information on the age of onset of the disease was also not available. 

**Figure 4:**
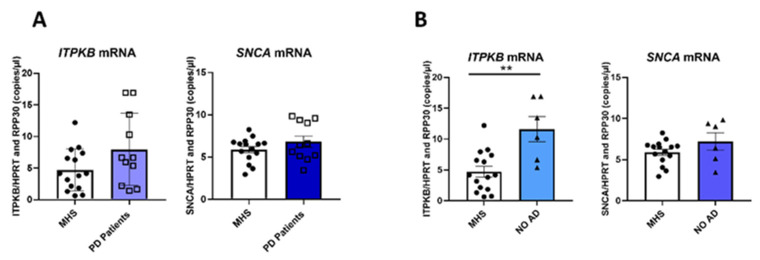
*ITPKB* and *SNCA* expression assessment in the cortex of PD patients and matched healthy controls. RNA was extracted from the cortex of 11 PD patients and 15 matched healthy subjects (MHS). *ITPKB* and *SNCA* mRNA levels were quantified using ddPCR. (**A**) Bar graphs show *ITPKB* (left) and *SNCA* (right) quantification in MHS and patients. (**B**) Patients who showed Alzheimer’s Disease co-morbidity were removed from the “PD patients’ group” (NO AD group). *ITPKB* (left) and *SNCA* (right) mRNA levels were compared with MHS. Mean ± s.e.m. (standard error of the mean) are shown. An unpaired two-tailed *T*-test with Welch’s correction was performed; ** *p* ≤ 0.01.

We measured mRNA levels of both *SNCA* and *ITPKB* in the samples. After cDNA synthesis, we quantified *SNCA* and *ITPKB* mRNAs by ddPCR (Figure 4). *HPRT* and *RPP30* were both used as reference genes for normalization of the results because their expression correlates (r = 0.94), which means that they have a similar mRNA level in the brain samples.

When we compared *ITPKB* mRNA levels in PD patients and controls (matched healthy control subjects = MHS) we observed an increase of 70% when pathology was present (Figure 4A, left panel). However, due to a wide variability of the kinase expression in the different samples, this increase is not statistically significant within this limited number of samples. For the *SNCA* mRNA, which shows a more homogenous expression between samples compared to the *ITPKB* mRNA, we did not observe any difference between the two groups (Figure 4A, right panel). We repeated the analysis by removing from the PD group those patients who showed Alzheimer’s Disease comorbidity (NO AD group) and compared *ITPKB* and *SNCA* mRNA levels with those from the control group. By removing the effect of possible confounding factors that might increase the gene expression variability, the *ITPKB* mRNA level is significant higher in the PD group without co-morbid AD when compared with healthy controls (*ITPKB* mRNA= 11.6 ± 2; 4.6 ± 0.86 copies/µL respectively, average ± SEM. Figure 4B, left panel). No difference was observed when we quantified *SNCA* mRNA levels between the two groups (Figure 4B, right panel). 

While the differences above were not always statistically significant, possibly due to limited sample numbers and inter-individual variability, we did observe a general increase of *ITPKB* mRNA in patients’ brains, which correlated with increased *SNCA* mRNA levels. We further analyzed the data by stratifying the patient data into two groups depending on *SNCA* expression levels. Based on the amount of *SNCA* mRNA present, PD patients whose mRNA levels were higher than the average in the healthy controls were included in the “High Expressing” (HE) group; conversely, patients whose *SNCA* mRNA levels were lower than the average in healthy controls were included in the “Low Expressing” (LE) group. 

**Figure 5:**
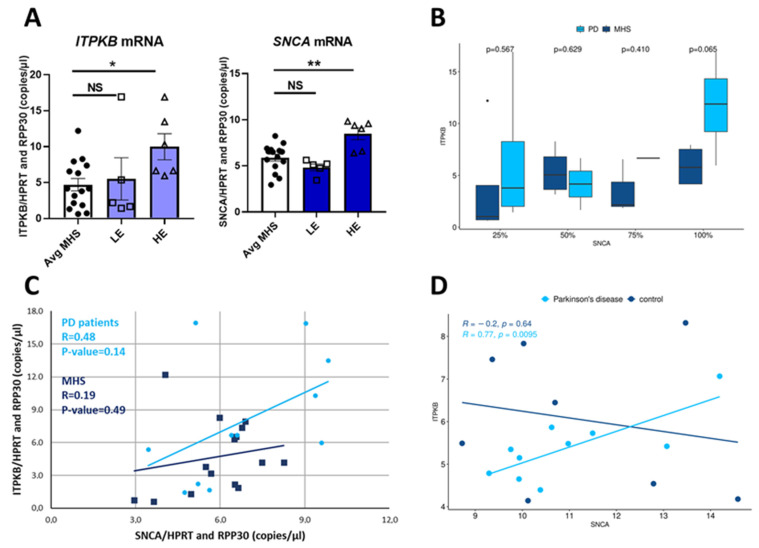
Correlation between *ITPKB* and *SNCA* expression in the cortex from PD patients and matched healthy controls. (**A**) *ITPKB* and *SNCA* mRNA levels in PD patients were compared with those in MHS. PD patients were divided in 2 groups: HE = *SNCA* mRNA level is >average *SNCA* level in MHS and LE = *SNCA* mRNA level is <average *SNCA* level in MHS. Bar graphs show *ITPKB* (left) and *SNCA* (right) mRNA quantification in MHS, LE and HE groups. Mean ± s.e.m. (standard error of the mean) are shown. An unpaired two-tailed *T*-test with Welch’s correction was performed. NS *p* > 0.05, * *p* ≤ 0.05, ** *p* ≤ 0.01. (**B**) Pairwise comparison cases vs. controls for each quartile of *SNCA* expression. A higher expression of SNCA better separates the cases from controls. (**C**) *ITPKB* and *SNCA* mRNA levels for each sample (patients and MHS) are plotted in the dot plots graph. The regression lines for the PD or MHS group are included. Pearson correlation analysis was performed for each group separately. (**D**) Correlation between expression for genes *SNCA* and *ITPKB* for 10 PD and 8 controls in dataset GSE020141 is significant for PD (light blue dots and regression line, R^2^ > 0.7, *p* < 0.01) while not significant for 8 controls (R^2^ = −0.2, *p* ≥ 0.65).

*ITPKB* and *SNCA* mRNA levels in the two groups were then correlated to each other with respect to quantification seen in the healthy controls (Figure 5).

*ITPKB* mRNA levels in the HE group, were significantly higher (2.1-fold) compared to controls while no difference was observed in the LE group (Figure 5A, left panel). In this analysis *SNCA* mRNA is also significantly increased by 44% in HE group compared to controls but does not change in the LE group (Figure 5A, right panel). To confirm the specificity of the effect observed, we quantified four additional genes, in part to compensate for possible differences in cell number between samples: *MAP2*, *RBFOX3* (*NeuN*), *RIT2* and *MAPT.* We did not observe any differences in *MAP2*, *NeuN* or *RIT2* expression in the presence of a high or low amount of *SNCA* mRNA (Supplementary Figure S1). The stability in the expression of the neural markers *NeuN* and *MAP2* indicates that there are no differences in the quantity of mature neurons or neurons and glia, respectively. RIT2 is a risk factor for PD [30] but no studies on its expression are currently available to indicate an increase in the cortex of PD patients. However, in the group in which *SNCA* is more expressed than in the average of the healthy controls (HE), we observed a significant increase (50%) also in *MAPT* expression (Supplementary Figure S1). This result is coherent with Ding and colleagues who reported that augmented level of α-synuclein influences TAU expression in SH-SY5Y cells treated with methamphetamine [31]. In addition, when we distributed *ITPKB* mRNA quantified in the PD patients into quartiles, based on *SNCA* expression in the control group, we see statistically significant differences at α = 0.1 comparing cases and controls falling into the highest quartile, suggesting the overall *SNCA* mRNA expression is higher in PD brains (Figure 5B). Thus, as increased *ITPKB* expression correlates with *SNCA* levels, we used a Pearson correlation analysis to examine the relationship between *SNCA* and *ITPBK* expression in the PD and control groups. In the PD group, the *ITPKB* mRNA level is directly correlated with *SNCA* amount while in the control group the correlation is weak (R = 0.48 and 0.19 in the PD and control groups, respectively, Figure 5C). Zheng et al. [32] conducted a genome-wide expression study on brain samples (substantia nigra) from 10 PD patients and 8 controls. We used the publicly available GEO dataset (GSE020141) to confirm the experimental observations from the correlation analysis in this second independent cohort dataset. After normalizing the gene expression and extracting the probes targeting genes *ITPKB* and *SNCA* we analyzed the correlation distribution of the two genes of PD against controls (Figure 5D). We found a similar pattern of correlation as the one observed in our dataset; in particular, an increase of *SNCA* expression in PD positively correlates with an increase in *ITPKB* expression, with Pearson’s R = 0.77 (*p* < 0.01). For the controls, we observed a weak negative correlation with *p* > 0.65 suggesting the R value is either not informative or no correlation could be observed. In conclusion, we interpret this data to show that in the cortex of PD patients, *ITPKB* expression is also augmented in the presence of increased levels of *SNCA* mRNA, and we replicated this observation using an external independent dataset. We quantified also ITPKB and α-synuclein protein levels in the same cohort but since we observed a high variability of protein levels, even in the control group, which did not match with the mRNA expression data, we decided not to include this data, or proceed further with an analysis of protein levels.

Thus, in the cortex of PD patients we confirmed what we observed in SK-N-SH cells, that overexpression of α-synuclein correlates with increased levels of *ITPKB* mRNA. 

## 3. Discussion

Alzheimer’s disease together with Parkinson’s disease are the most common neurodegenerative pathologies worldwide. Life expectancy for PD patients is increasing and consequently, caregivers’ efforts to improve their quality of life are also increased, as well as the healthcare costs for the community [33]. For these reasons, greater knowledge of the mechanisms at the basis of PD and its progression is of extreme importance, to increase the chances of finding a way to ameliorate the clinical prognosis in the patients.

So far, more than 20 genes and over 90 genetic risk variants have been associated with the insurgence and or progression of familial and sporadic PD [30,34], indicating that the disease is, at least in part, driven or modified by genetic factors. The identification of several of these factors in PD converge to common pathways such as neuronal networking, vesicular trafficking, autophagy, mitochondrial metabolism and the lysosomal pathway [12]. Of interest, rare variants located in different genes can be co-inherited in about 17% of familial/sporadic PD, pointing again to a polygenic contribution to the pathology [35]. Finally, the clinical phenotype can vary between PD patients that carry the same mutations [11], either because of environmental factors or additional genetic risk factors. 

Here, we evaluated one such PD genetic risk factor, *ITPKB*, in different *SNCA* backgrounds to understand possible correlations between the well-known A53T and A30P mutations or *SNCA* gene multiplication and this specific kinase. We selected ITPKB for the following reasons: firstly, we have observed that α-synuclein overexpression prevents agonist-dependent calcium increase in the cytosol by blunting the PLCβ signaling pathway [24]. Because ITPKB regulates calcium homeostasis, we postulated whether it may also be dysregulated. Secondly, its role in reducing the formation of α-synuclein aggregates was proposed [22]. Thirdly, ITPKB was shown to act as a risk factor in AD [18,36]. While there is no concordance in the direction of the ITPKB effect in PD or AD, this kinase has a reported impact on both of the most common neurodegenerative diseases. Moreover, to our knowledge, no studies are currently available describing the interaction between ITPKB and wild-type α-synuclein or its mutated isoforms.

We initially validated the role of ITPKB in modulating α-synuclein toxicity in vivo in a *Drosophila melanogaster* model ectopically expressing human α-synuclein in the nervous system. By artificially reducing the expression of *IP3K2*, the closest fly ortholog for human ITPKB, we could increase the lifespan and climbing activities in these flies. In this way, we validate the synergy between ITPKB and α-synuclein to modulate PD in a model system. We also provide evidence that, at least in this PD model in which the IP3K2 protein is still present even if at low dosage, ITPKB may act as a risk factor for the pathology. 

Then, we characterized *ITPKB* expression in human SK-N-SH cells overexpressing wild-type α-synuclein or its mutated isoforms. We selected these cells because they are widely used in the field, and we had previously engineered them to overexpress α-synuclein and A53T/A30P mutants. In these cells, α-synuclein overexpression prevented the Ca^2+^ release from the ER. Since ITPKB, by converting IP3 into IP4, reduces the release of Ca^2+^ from the ER pool, we reasoned that it may represent a downstream effect on the ITPKB pathway in these α-synuclein-overexpressing models [24]. The presence of high levels of α-synuclein impacted ITPKB expression at both mRNA and protein levels, independently of the *SNCA* mutations. The ITPKB increase was specifically correlated with α-synuclein increase because when we quantified its mRNA level in SH-SY5Y cells overexpressing another PD risk factor, LRRK2 or LRRK2 G2019S, the kinase transcription was not affected or was reduced. Together with SK-N-SH cells overexpressing α-synuclein, these cells also represent a good model for PD because when LRRK2 G2019S was overexpressed, we observed the accumulation of phosphorylated α-synuclein and the formation of inclusion-like structures [25]. Of note, in SH-SY5Y cells overexpressing LRRK2 or its hyperactivated isoform, the total amount of α-synuclein did not change compared to SH-SY5Y cells (reported in [25]), so, in this model, the ITPKB decrease is induced by LRRK2 G2019S overexpression. Because ITPKB was differently regulated in these two cellular models, we deduced that α-synuclein may enhance *ITPKB* transcription. By analyzing the promoter region of *ITPKB* (NM_002221) using the Jaspar database (https://jaspar.genereg.net/ [37], interrogated on 14 October2022) we identified the binding site for the transactivating transcription factor 1 (sp1) in the proximity of the putative promoter region. The monoamine oxidase A (MAOA), one of the enzymes regulating dopamine metabolism, also contains the sp1 binding domain in its promoter [38]. Overexpression of mice α-synuclein or the A53T mutant in mice neuroblastoma cells lines increases sp1 expression which, through the binding with the promoter, enhances MAOA expression [38]. This mechanism could also explain the increase of ITPKB in the presence of high level of α-synuclein observed in our experiments. 

Next, we analyzed whether the overexpression of wild-type or A53T or A30P α-synuclein isoforms may influence ITPKB localization and thus have an impact on its function, i.e., the regulation of Ca^2+^ homeostasis. It was already reported that A53T or A30P α-synuclein mutants are enriched in the nucleus where they reduce histone H3 acetylation [28,29]. We also observed the enrichment of mutated α-synuclein in the nucleus of SK-N-SH cells compared to the wild-type isoform. In this model, ITPKB expression was increased in both compartments, where each α-synuclein isoform was also overexpressed. Of interest, when A53T and especially A30P mutant isoforms are expressed, we observed the increase of the kinase at slightly higher levels compared to the wild-type in the cytoplasm. This difference may be due to the increased ability of A53T and A30P to induce transcription when mislocated in the nucleus or due to the reduced level of wild-type isoforms compared to A53T and A30P isoforms. Higher levels of ITPKB in the cytoplasm may lead to an augmented conversion of IP3 to IP4, thus to a reduction of IP3R activity, that leads to a reduction of calcium release from the ER and consequently to a depleted uptake in the mitochondria. In the future, experiments devoted to the characterization of calcium homeostasis in the presence of the A53T and A30P isoforms of α-synuclein should clarify this aspect. In addition, in further experiments we will evaluate if ITPKB enrichment in the cytoplasm is due the enrichment in specific organelles like the endoplasmic reticulum. 

Since we observed a specific increase of ITPKB expression in the presence of increased α-synuclein in human neuroblastoma cell lines, we selected the brain cortex of PD patients, a region also affected in the disease, to establish whether this correlated regulation of *ITPKB* also occurs in vivo. In our cohort, no differences were observed in *SNCA* expression between PD patients and controls while *ITPKB* seems to be more expressed in the patients’ group compared to controls. 

According to Braak stage theory, in PD, α-synuclein deposition reaches the cortical area in the later stages of the disease whilst the Aβ deposition is observed first in the neocortex of AD patients (reviewed in [39]). According to this scheme, cortex from PD Patients with severe AD would be more affected compared to those from PD patients with early Alzheimer’s [39]. Since ITPKB is involved in both pathologies, its expression could be influenced also by AD severity in the patients. When those samples with a comorbidity of Alzheimer’s Disease with an unknown Braak stage were excluded from the patient group, the *ITPKB* increase in this PD group became statistically significant. This may be explained because AD co-morbidities reported in patients are present at different levels of severity and could represent a confounding factor. Moreover, the expression of several PD risk genes, including *ITPKB*, may change in different regions of the brain in PD [40], as well as in AD [41] patients and due to the limited size of our cohort, subtle differences can be masked by grouping all the patients together. To analyze *ITPKB* and *SNCA* correlation in more detail, we grouped the patients in our cohort based on *SNCA* level in high- and low-expressing subgroups and compared *ITPKB* levels in these groups and with the control group. Those patients whose *SNCA* level was higher than the average in the control groups showed also significant *ITPKB* increases of approximately 2.1-fold compared to healthy controls. The same effect was observed with *MAPT,* for which mRNA levels were increased in patients with high SNCA levels but not with the neuronal markers *MAP2* or *NeuN* or the PD risk factor *RIT2*. Finally, when we measured the relation between *ITPKB* and *SNCA* expression we observed a positive correlation only in the patients’ group and in a public dataset obtained from the substantia nigra, thus reinforcing our observations at least at the transcription level.

Therefore, our results show that the PD genetic risk factor gene *ITPKB* is upregulated in human neuroblastoma cell lines overexpressing wild-type or mutated α-synuclein and in cortex from PD brains. We tested whether α-synuclein may upregulate ITPKB expression by modulating its transcription. Moreover, in our experiments, in vivo ITPKB reduction ameliorates PD phenotypes in *Drosophila melanogaster* expressing human α-synuclein. These results are coherent with what was already suggested for AD, in which amyloid-β peptide formation is increased by *ITPKB* expression [19]. We have also shown that *ITPKB* is downregulated when LRRK2 G2019S is overexpressed, and thus the upregulation may be specific for α-synuclein. These results are coherent with what was observed in *Drosophila* expressing LRRK2 G2019S in the central nervous system, in which mitochondrial calcium uniporter (MCU) expression is downregulated. The authors proposed that MCU reduction is protective because it avoids mitochondrial calcium overload due to the Ca^2+^ increase in the cytosol, which, once entered, would lead to ATP overproduction and caspase C activation [42]. Of interest, ITPKB is also involved in Ca^2+^ homeostasis and Apicco [22] proposed that it acts by inhibiting Ca^2+^ release from ER and its uptake into the mitochondria via MCU. Thus, we propose that, depending on the mutation carried by the patients (i.e., *LRRK2* or *SNCA*), and depending on the specific cell type, ITPKB may contribute to PD with different mechanisms involving calcium homeostasis, and thus act as a protective or risk factor. 

Our data were produced in two different models in which we focused on the relationship between ITPKB and α-synuclein, but these models do not include dopaminergic neurons or tissues originating from the substantia nigra *pars compacta* (SNpc) of PD patients in which the role of ITPKB should be investigated in more detail. If the role of ITPKB as risk factor for PD will be further confirmed, inhibition of this kinase activity may offer alternative therapeutical approaches for PD patients, especially those in which *SNCA* multiplication or A53T/A30P have been identified. Of importance, it was shown that *Itpkb* inhibition by the chemical compound GNF362 in rats reduced autoimmune arthritis by decreasing lymphocyte T survival [43] and the graft versus host disease (GVHD) lethality, a pathological condition that stimulates immune system response after transplantation [44]. Thus, GNF362 may be a viable inhibitor of ITPKB, and should be tested in dopaminergic models of PD as to whether it can be neuroprotective, and in finding wider applications to the neurodegenerative field.

## 4. Materials and Methods

**Figure 6:**
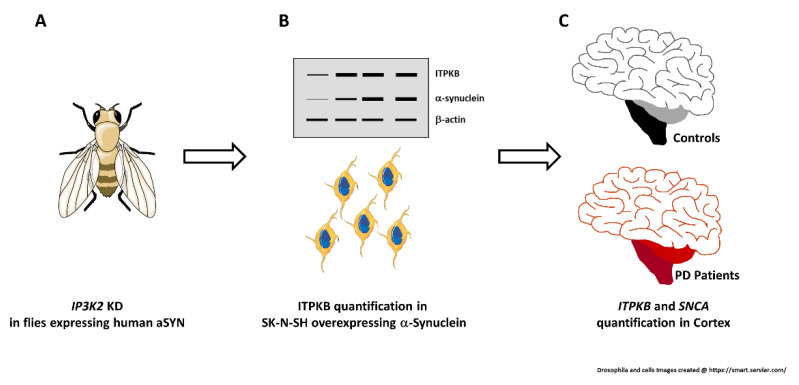
Experimental workflow. IP3K2 expression was reduced in flies overexpressing human α-synuclein. Geotaxis and lifespan PD-related phenotypes were measured (**A**). ITPKB was quantified in SK-N-SH cells overexpressing wild-type or mutated A53T or A30P α-synuclein by western blot (**B**, top). ITPKB protein level was measured also in the cytoplasm and in the nucleus of cells, separately (**B**, bottom). *ITPKB* and *SNCA* mRNA levels were quantified in cortex from PD patients and controls (**C**).

The description of the experimental workflow is shown in Figure 6. 

### 4.1. Cell Lines

Human neuroblastoma cell lines, SK-N-SH, were cultivated in RPMI (L0501, Biowest, Voden, Monza; Italy) supplemented with 10% FBS (F2442, Sigma-Aldrich, Milan, Italy), 1% sodium pyruvate (S8636, Sigma-Aldrich, Milan, Italy), 1% non-essential amino acids (R7131, Sigma-Aldrich, Milan, Italy) and 1% pen/strep solution (L0022, Biowest, Voden, Monza, Italy). SK-N-SH cells stably overexpressing wild-type or A53T/A30P α-synuclein were previously generated in our lab [24]. G418 (10131-027, Life technology, Thermo Fisher Scientific, Milan, Italy), at a final concentration of 450 µg/mL, was added to the culture for wt or mutated α-synuclein selection. Human neuroblastoma cell lines, SH-SY5Y, were cultivated in DMEM high glucose (L0103, Biowest, Voden, Monza, Italy) supplemented with 10% FBS (F2442, Sigma-Aldrich, Milan, Italy) and 1% pen/strep solution (L0022, Biowest, Voden, Monza; Italy). SH-SY5Y stable-expressing LRRK2 or LRRK2 G2019S were generously donated by Evy Lobbestael and Veerle Baekelandt (Laboratory for Neurobiology and Gene Therapy, Department of Neurosciences, Leuven Brain Institute, KU Leuven, Leuven, Belgium) and cultured in DMEM high glucose (L0103, Biowest, Voden, Monza, Italy) supplemented with 15% FBS (F2442, Sigma-Aldrich, Milan, Italy) and non-essential amino acids (M7145, Sigma-Aldrich, Milan, Italy). Gentamicin sulfate (50 µg/mL, 5245 Biomed reagents, Hilden, Germany) and Hygromycin B (200 or 400 µg/mL for LRRK2 or LRRK2 G2019S, 10687010 Gibco, Thermo Fisher Scientific, Milan, Italy) were added for clone selection. Cells were maintained in culture at 37 °C at 5% CO_2_

### 4.2. Brain Tissue Samples

Autopsy brain tissues were obtained from the Cambridge Brain Bank Laboratory (Cambridge, UK). The Cambridge Local Ethical Review Committee approved the collection and storage of tissue, as well as all research procedures carried out in the present study (REC reference: 10/H0308/56). The Local Ethical Review Committee of the Bolzano Hospital also approved the research procedure used in this study (Parere Nr.0045821/2016, Comitato Etico dell’Azienda sanitaria di Bolzano). A total of 26 brains were examined, of which 11 were obtained from patients diagnosed with Parkinson Disease in life and 15 were obtained from people without neurological or neurodegenerative disorders and used as control subjects. In Table 1 are reported the demographic details. The postmortem interval (PMI) was taken as the time between witnessed death and the freezing of tissue after autopsy. Medical records from control subjects were reviewed to determine whether they had an active neurodegenerative disorder in the period before death and/or earlier in life. None of the control subjects had a clinical history of neurodegenerative disorders. Prefrontal cortex was dissected from brains at the time of autopsy. Samples from Brodmann area (BA) 10 were taken from the portion of the middle frontal gyrus rostral to a vertical cut 2 cm posterior to the apex of the frontal pole and snap-frozen. Full thickness cortical samples, dissected free of white matter, were crushed and lysed in Trizol (15596026; Thermo Fisher Scientific, Milan, Italy) to extract total RNA as described in the “Protein extraction and Western blot” session.

### 4.3. RNA Extraction, cDNA Synthesis and Droplet Digital PCR (ddPCR)

Total RNA was extracted from cells using RNeasy Plus Mini Kit (74136; Qiagen, Milan, Italy), following the manufacturer’s instructions. Briefly, pellets were lysed in RLT buffer supplemented with β-mercaptoethanol (M3148; Sigma-Aldrich, Milan, Italy) and on-column extraction was performed. DNAse treatment (79256; Qiagen, Milan, italy) was carried out to remove DNA contamination. Total RNA was quantified using the Qubit RNA HS Assay Kit (Q32852, Q32855; Thermo Fisher Scientific, Milan, Italy). Then, 1 µg of RNA was used for retrotranscription using the SuperScript VILO cDNA Synthesis Kit (11754-250; Thermo Fisher Scientific, Milan, Italy) following the manufacturer’s instructions. To quantify *ITPKB* and *SNCA* expression, 1 ng of cDNA was used for ddPCR experiments using Probe Supermix (No dUTP) (1863025; Bio-Rad Laboratories, Milan, Italy). A predesigned assay was used to amplify *ITPKB* (HsPT584772564, IDT, Tema Ricerca, Bologna, Italy). *RPP30* (dHsaCP2500350, Bio- Rad Laboratories, Milan, Italy) and *HPRT* (dHSaCPE5192872, Bio Rad Laboratories, Milan, Italy) were used to normalize the RNA concentration among samples. Assay information is reported in Table S1. Droplets were generated using the QX100 Droplet Generator (Bio-Rad Laboratories, Milan, Italy). The thermal profile for PCR amplification is the following: 10 min at 95 °C, 40 cycles consisting of 30 s at 94 °C followed by an annealing and extension step at 57 °C for 1 min, and a final step of 10 min at 98 °C. Droplets were analyzed using Bio-Rad QX Manager v. 1.2 and the concentration was calculated according to software’s instructions. 

Total RNA from cortex was extracted using a TeSeE PRECESS 24 homogenizer (3591070; Bio-Rad Laboratories, Milan, Italy) and lysis was done in a cold room at 4 °C; the homogenizer and centrifuge were pre-cooled. Sterile 2 mL grinding tubes with a screw cap were filled with 2.6–2.9 g zirconium beads ø 1 mm (Z763780-50EA, Bead Bug prefilled tubes, Sigma-Aldrich, Milan, Italy), 1 mL TriZol 4 °C reagent (15596026; Thermo Fisher Scientific, Milan, Italy), and then were weighed and put on ice. In a sterile urine sample container (Pharmacy) kept on dry ice, a small piece of frozen tissue was picked with a biopsy punch ø 2 mm (BPP-20F; KAI Medical, Solingen, Germany) from the samples stored in liquid N_2_ and immediately transferred in the TriZol-filled tubes. Tissue was homogenized at 5500 rpm for 40 s followed by 60 s rest, repeating once. Tubes were weighed a second time and put in a centrifuge at 4 °C for 5 min at 14,000× *g*. Total RNA was purified using the Direct-zol RNA Miniprep Kit (R2052; Zymo Research, Irvine, CA, USA) following the manufacturer’s instructions. Briefly, 2 × 900 µL supernatant was carefully transferred to 2 new tubes per sample filled with 900 µL of absolute ethanol each and the content was mixed by pipetting up and down carefully. The obtained mix was loaded on a spin column in 3 rounds. After each load, the column was centrifuged briefly, and eluate was discarded. A pre-wash round was done followed by a 15 min incubation with DNase I at RT. Three more wash rounds were performed and the RNA was eluted in sterile water. RNA quantification and cDNA synthesis were performed as described before with the only exception that 200 ng of RNA were retrotranscribed. For ddPCR quantification, an *SNCA* probe (HsPT58912923, IDT, Tema Ricerca, Bologna, Italy) was used together with the probes described before. 

### 4.4. Protein Extraction and Western Blot

Cells were lysed in RIPA buffer (89900, Thermo Fisher Scientific, Milan, Italy) supplemented with protease (04693124001, Roche, Sigma-Aldrich, Milan, Italy) and phosphatase inhibitors (04906837001, Roche, Sigma-Aldrich, Milan, Italy). Lysates were sonicated for 10 sec at 10% intensity using Sonoplus GM 2070 instrument (Bandelin, Berlin, Germany) and centrifuged at 10,000× *g* for 10 min at 4 °C to remove genomic DNA. Supernatant was recovered to quantify proteins using the Pierce^TM^ Bicinchoninic Acid (BCA) Protein Assay Kit (23225, Thermo Fisher Scientific, Milan, Italy) following the manufacturer’ instructions. 

Then, 30 µg of proteins was diluted with NuPage^TM^ LDS sample buffer (NP0007, Invitrogen, Thermo Fisher Scientific, Milan, Italy), containing 50 mM dithiothreitol (DTT), heated at 95 °C for 5 min and loaded in NuPAGE^TM^ 4–12% Bis-Tris Protein Gels (NP0335, Invitrogen, Thermo Fisher Scientific, Milan, Italy). Proteins were transferred onto a 0.2 µm polyvinylidene difluoride (PVDF) membrane (1620177, Bio-Rad Laboratories, Milan, Italy) and blocked for 1 h at room temperature in 5% non-fat dried milk (70166 Sigma-Aldrich, Milan, Italy) dissolved in TBS (TRIS 50 mM, NaCl 150 mM, pH 7.5)–Tween 0.1%. Membranes were stained with anti ITPKB (1:2000, 12816-1-AP, Proteintech, DBA, Milan, Italy), anti α-synuclein (1:2000, MAB5383, Abnova, Taipei City, Taiwan) or anti β-actin (1:8000, A-5316, Sigma-Aldrich, Milan, Italy). Chemiluminescence images were acquired using the Clarity Western ECL Substrate (Bio-Rad Laboratories, Milan, Italy, #1705061) detection reagent.

### 4.5. Immunocytochemistry (ICC) and Images Acquisition

For immunocytochemistry (ICC), SK-N-SH cells were fixed with 4% paraformaldehyde (158127; Sigma-Aldrich, Milan, Italy), then permeabilized with Triton X-100 (T8787; Sigma-Aldrich, Milan, Italy), blocked with normal donkey serum (ab7475, abcam, Cambridge, UK), and incubated with the primary antibodies overnight at 4 °C (rabbit anti ITPKB, 12816-1-AP Proteintech, DBA, Milan, Italy, 1:1500 and mouse anti α-synuclein, Abnova, Taipei city, Taiwan, MAB5383, 1:1000). The following day, after washing with PBS, coverslips were incubated with secondary fluorescent antibodies for 2 h at RT (Alexa Fluor 488 or Alexa Fluor 555, A21206 and A31570 Thermo Fisher Scientific, Milan, Italy), washed with PBS and mounted with DAKO Fluorescence Mounting Medium (S3023; Agilent technologies, Santa Clara, CA, USA). Visualization was performed using a Leica SP8-X confocal microscope system. Using Imaris analysis software (Oxford Instruments, Abingdon, UK), the intensity of the ITPKB staining was quantified in 3D stacks of images that had been acquired with identical, non-saturating settings. After modeling the nucleus and the cytoplasm, the mean ITPKB intensity of either whole cells or nuclei/cytoplasm compartments was calculated on a 0–255 scale. Five samples were used for each case, with each sample consisting of a field with roughly 20 cells, for which intensity values were used.

### 4.6. Fly Husbandry

Flies were reared on a standard diet of corn meal, yeast and molasses at 18–25 °C and 70% humidity under a 12 h:12 h light:dark cycle. The PD model (elavGal4; +/CyOtubgal80; UAS-h-αSyn) and its control (elavGal4; +/+; UAS-mCD8GFP) were generated in the Neely Lab using lines (458, 8146, 9491) from the Bloomington Drosophila Stock Center (BDSC). UAS-IR transgenic fly and w1118 control lines were obtained from the Vienna Drosophila Resource Centre (VDRC) RNAi library [45]. The IP3K2 RNAi hairpin used was 102730. All experimental flies tested in this study were male and an isogenic w1118 background was maintained throughout all experiments.

### 4.7. Locomotor Function and Lifespan

Three vials of 15 male flies were collected per genotype. The number of dead flies was counted every 2–3 days when flies were transferred to new medium. Locomotor function was assessed once per week throughout the fly’s lifespan, as described previously [46,47]. Briefly, flies were gently tapped to the bottom of the vial and given ten seconds to climb a distance of five centimeters. The number of flies that failed to reach the distance was then recorded. Each vial was tested three times at each time point with 30 s rest. Climbing significance was calculated using a one-way ANOVA with a post-hoc Dunnett test at each time point and area under the curve analysis. Lifespan data for each screening experiment was analyzed using a log-rank Mantel-Cox test. 

### 4.8. Statistical Analysis

All data are expressed as Mean ± s.e.m. (standard error of the mean). Statistical significance was calculated using a one-way ANOVA with Bonferroni’s correction for multiple comparisons or the Student’s *t*-test with or without Welch’s correction for unequal distributions. The significance level was indicated as NS, *p* < 0.05 * *p* ≤ 0.05, ** *p* ≤ 0.01. and **** *p* ≤ 0.0001. Linear correlations were measured using Pearson correlation coefficient (R).

## 5. Conclusions

The present study analysed the correlation between the expression of α-synuclein and ITPKB, two risk factors for PD, in human neuroblastoma cell lines and in cortex from PD patients. We found that when α-synuclein level is higher, ITPKB expression is increased. Increased α-synuclein expression could mimic the triplication of the *SNCA* gene found in some PD patients. On the contrary, A53T or A30P, other well-known PD mutations in α-synuclein, did not exert any different effect on ITPKB expression compared to the wild-type, but did seem to enrich the kinase especially in the cytoplasmatic compartment, which would have consequences for calcium homeostasis in this compartment. Finally, our data on flies show that ITPKB reduction is beneficial to rescue the survival phenotype in animals expressing human α-synuclein. Additional experiments in the dopaminergic neurons in the substantia nigra *pars compacta* (SNpc) in the midbrain should be performed to reveal the contribution of ITPKB in regulating Ca^2+^ homeostasis. Once this information is available, testing as to whether the modulation of ITPKB expression or activity ameliorates the PD condition could be undertaken. 

## Figures and Tables

**Table 1:** Demographic and clinical characteristics of the samples included in the study. In the first column, P1 to P11 indicate the PD patients while C1 to C15 are the healthy control subjects. Age at the death and sex (F = female, M = male) are reported in the second and third columns, respectively. Co-morbidity with Alzheimer’s disease (AD) is reported in the fourth column while the presence of Lewy bodies (LB) is shown in the fifth column. When available, postmortem interval (PMI) is indicated in the last column. NA = not available.

Patient ID	Age at Death (Years)	Sex	AD	LB	PMI (h)
P1	73	F	No	Diffuse	22.3
P2	81	F	No	No	78.3
P3	86	F	Early Alzheimer’s	Diffuse	NA
P4	84	M	Alzheimer’s	No	NA
P5	77	M	Early Alzheimer’s	Yes	7
P6	71	M	Alzheimer’s	No	NA
P7	70	F	No	Diffuse	8
P8	79	F	Alzheimer’s type pathology	No	NA
P9	86	M	No	Diffuse	NA
P10	78	M	No	No	23
P11	85	F	No	Yes	13.3
C1	76	M	-	-	23.3
C2	88	M	-	-	NA
C3	73	M	-	-	13
C4	69	W	-	-	54
C5	69	M	-	-	55
C6	85	F	-	-	24
C7	61	M	-	-	28.3
C8	61	F	-	-	71
C9	78	M	-	-	77
C10	76	F	-	-	6.5
C11	72	M	-	-	38.3
C12	76	F	-	-	24
C13	76	M	-	-	41.3
C14	72	F	-	-	64.3
C15	76	M	-	-	32.3

## Data Availability

Not applicable.

## Supplementary Materials

The following supporting information can be downloaded at: https://www.mdpi.com/article/10.3390/ijms24031984/s1.
