# Supplementary material for: Increased Levels of the Parkinson’s Disease-Associated Gene ITPKB Correlate with Higher Expression Levels of α-Synuclein, Independent of Mutation Status
Source: Int J Mol Sci. 2023 Jan 19;24(3):1984. doi: 10.3390/ijms24031984 (PMC9916293; doi:10.3390/ijms24031984)
Supplement: Supplementary file 1 [file ijms-24-01984-s001.zip › ijms-2100851-supplementary.pdf]

## Droplet digital PCR (ddPCR)

To evaluate gene expression in the cortex, ddPCR experiments were performed using 0,2 ng of cDNA to amplify *MAPT* (HsPT5839989875, IDT, Tema Ricerca, Bologna, Italy) or 1ng of cDNA to amplify *RIT2* (HsPT58196183, IDT, Tema Ricerca, Bologna, Italy) *RFOX3* (HsPT582776427, IDT, Tema Ricerca, Bologna, Italy) and *MAP2* (HsPT5820103440, IDT, Tema Ricerca, Bologna, Italy). Primer sequences are reported in Table S1. Probe Supermix (No dUTP) (1863025; Bio-Rad Laboratories, Milan, Italy) was used for amplification using the following thermal profile: 10 minutes at 95°C, 40 cycles consisting of 30 seconds at 94°C followed by an annealing and extension step at 57°C for 1 minute, and a final step of 10 minutes at 98°C. Droplets were analyzed using Bio-Rad QX Manager v. 1.2 and transcript expression was quantified according to the software´s instructions.

## Supplementary Figures

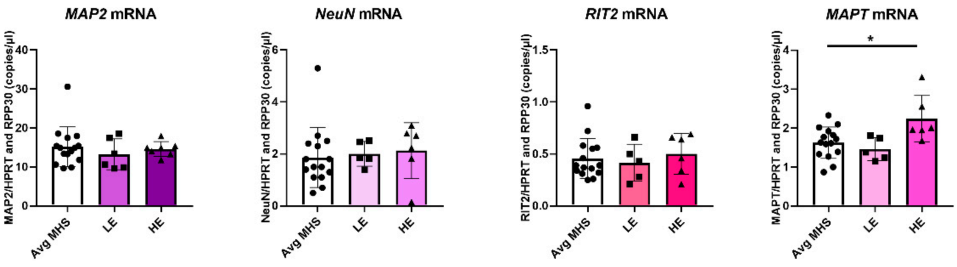


**Supplementary Figure S1:** Gene expression assessment of additional genes in the cortex from PD patients and matched healthy controls. ddPCR was used to quantify *MAP2*, *RBFOX3* (NeuN), *RIT2*and *MAPT* mRNA level in the RNA extracted from 11 PD Patients and 15 Matched Healthy controls. Patients were divided in two groups according to *SNCA* mRNA level. Patients whose *SNCA* level is >average *SNCA* level in MHS belong to „HE group" while those with *SNCA* level < average *SNCA* level in MHS belong to „LE group". Bar graphs show *MAP2, NeuN, RIT2* and *MAPT* mRNA levels. mRNA levels quantified in PD patients and healthy controls were compared between HE and LE groups and with the MHS. Mean ± s.e.m. (standard error of the mean) are shown. 1-way ANOVA with Bonferroni's correction for multiple comparison to calculate statistical significance. NS P>0.05, * P≤0.05.

**Table S1:** List of primers used for ddPCR experiments. Primer sequences for each assay are reported in the table. Assays for which this information was not available (NA), the sequence for assay´s amplicon is reported (MIQE).

| Target gene | Assay name | Primer1 (5´→3´) | Primer2 (5´→3´) | Amplified sequence (MIQE) |
| --- | --- | --- | --- | --- |
| ***ITPKB*** | HsPT584772564 | ATGGACTGCAAGATGGGAATC | GTCCACCTCGATCATCTTCTG | - |
| ***MAPT*** | HsPT5839989875 | AAGAGCCGCCTGCAGAC | AACTGGTTTGTAGACTATTTGCAC | - |
| ***RIT2*** | HsPT58196183 | GCAGTTTATTAGTCATCAGTTCCC | AGCAGTGTCCAAGATGTCC | - |
| ***RFOX3*** | HsPT582776427 | CGGTCGTGTATCAGGATGGA | GCCGTAACTGTCGCTGTAG | - |
| ***MAP2*** | HsPT5820103440 | ACCTTCCTCCATTCTCCCT | ACCACTCTTCCCTGCTCT | - |
| ***RPP30*** | dHsaCPE5038241 | NA | NA | TATTAATGTGGCGATTGACCGAGGCCTGGCTTTTGAACTTGTCTATAGCCCTGCTATCAAAGACTCCACAATGAGAAGGTATACAATTTCCAGTGCCCTCAATTTGATGCAAATCTGCAAAG |

| | | | | |
|---|---|---|---|---|
| ***HPRT*** | dHSaCPE5192872 | NA | NA | ACTGGCAAAACAATGCAGAC TTTGCTTTCCTTGGTCAGGCAG TATAATCCAAAGATGGTCAAG GTCGCAAGCTTGCTGGTGAAA AGGACCCCACGAAGTGTTGGA TATAAGCCAGACTTTGTT |
